# The state of the journal: the *Journal of the Medical Library Association* in 2020

**DOI:** 10.5195/jmla.2020.1075

**Published:** 2020-10-01

**Authors:** Katherine G. Akers, Jill Barr-Walker, Kathleen Amos

**Affiliations:** 1 JMLA@journals.pitt.edu, Editor-in-Chief, *Journal of the Medical Library Association*; 2 jbwjmla@gmail.com, Associate Editor, *Journal of the Medical Library Association*; 3 kamos@phf.org, Associate Editor, *Journal of the Medical Library Association*

## Abstract

As the premier journal in health sciences librarianship, the *Journal of the Medical Library Association* (*JMLA*) continuously strives to publish high-quality work that advances research and practice and to provide irreplaceable value for readers, authors, and reviewers. This editorial reflects on the state of *JMLA* in 2020 by describing our editorial team and volume of submissions, highlighting recent initiatives that strengthen the journal's position in the profession, and sharing future plans to enrich *JMLA*'s content and promote open science. Committed to ending structural racism and other inequities in the field, we also issue an ongoing call for submissions pertaining to social justice and critical perspectives on health sciences librarianship.

In 2020, the *Journal of the Medical Library Association* (*JMLA*) continues to be the premier journal in health sciences librarianship. This appears to be due to a virtuous cycle. We actively strive to improve the experiences of our readers, authors, and reviewers; enforce increasingly high standards for the work we publish; and adopt policies and practices that keep the journal at the cutting edge of scholarly publishing. In return, authors continue sending us their best work, which allows us to set the bar even higher. To acknowledge the vast number of people who contribute to the journal's success and to shine light on some of our internal functions, we reflect on the past four years of *JMLA*'s publishing activity and initiatives and offer some glimpses into the journal's future.

## EDITORIAL TEAM

Presently, the *JMLA* editorial team includes an editor-in-chief, two associate editors, a managing editor, nine section editors, a social media editor, and an approximately twenty-five-member editorial board. Apart from the managing editor, who is employed by the Medical Library Association (MLA), all members of the *JMLA* editorial team are volunteers and do not get paid for their work. For the editor-in-chief and associate editors, this work includes screening submitted manuscripts, selecting and communicating with peer reviewers, synthesizing peer-reviewer comments, making editorial decisions, and often conducting additional rounds of editorial review to improve the writing and the presentation of data. For the section editors, the work includes soliciting manuscript submissions, working with authors to produce manuscripts that meet *JMLA* guidelines, and writing their own pieces. For editorial board members, this includes carrying a heavy peer-review load, providing feedback on journal administrative and policy decisions, and serving on task forces dedicated to improving key aspects of the journal. Without the extensive efforts of this volunteer team, *JMLA* in its current form would not exist.

## NEW PUBLISHING PLATFORM

In 2017, *JMLA* began publishing via the Open Journal System (OJS) journal management and publishing platform, hosted by the University of Pittsburgh, to take advantage of its relatively easy and robust work flows for manuscript submission, peer review, editorial decision making, and copyediting. Overall, we have been pleased with this platform and customer support and appreciate that its features have improved the efficiency and transparency of the review and publishing processes and enhanced the reading experience. All *JMLA* articles, including those published before 2017, continue to be available through PubMed Central.

## NUMBER OF SUBMISSIONS AND ACCEPTANCE RATE

**Figure 1:**
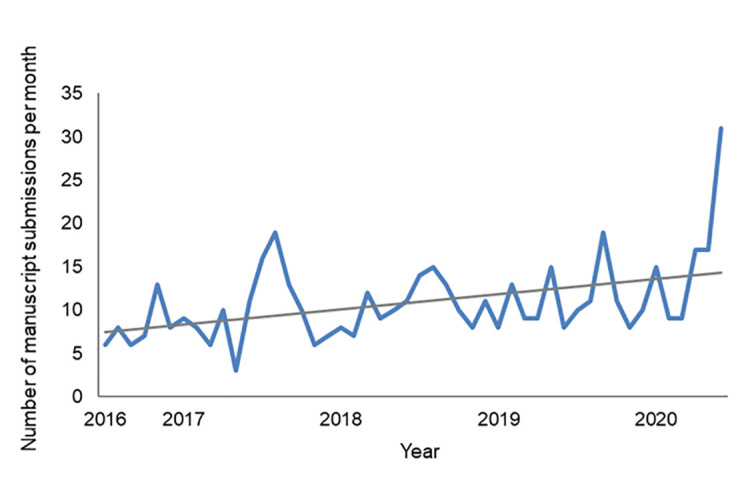
Number of submitted manuscripts per month across years

**Figure 2:**
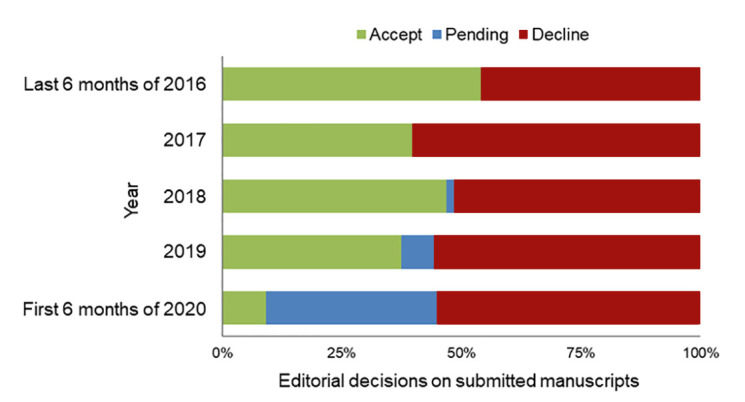
Percentage of submitted manuscripts that are accepted, declined, or pending an editorial decision across years

Between 2016 and 2020, *JMLA* received an average of approximately 11 unsolicited manuscripts per month, with a trend toward an increasing submission rate over time (Figure 1). Interestingly, shortly after the COVID-19 virus infiltrated North America, we experienced a notable increase in the number of submissions, with an unprecedented 31 manuscripts received in June 2020. Fewer than half (~40%–45%) of submitted manuscripts are ultimately accepted after peer and/or editorial review (Figure 2). Published issues of *JMLA* comprise approximately 50% peer-reviewed articles (i.e., knowledge syntheses, original investigations, case reports, special papers), 20% book and resource reviews, 15% commentaries and editorials, and 15% other articles (e.g., virtual projects, history matters, obituaries, MLA association records).

## EXPANSION OF PEER-REVIEWER POOL

We have drastically expanded *JMLA*'s peer-reviewer pool to include expert librarians and information professionals, information scientists, and health professionals regardless of their role with the journal or their membership in MLA. At present, the *JMLA* peer-reviewer database contains nearly 600 individuals. Deeply appreciative of their time and thoughtfulness, we publicly recognize our reviewers in annual editorials, the most recent of which reports that 220 individuals reviewed for *JMLA* in 2019 [1]. We believe this expansion of the *JMLA* peer-reviewer pool has markedly improved the rigor with which submitted manuscripts are evaluated. To support this group of reviewers and meet a desire for peer-reviewer training for health sciences librarians, we presented an MLA webinar, “Everything You Ever Wanted to Know about Peer Review: For Reviewers and Authors,” in June 2020 [2], and additional resources and training opportunities for building peer reviewers' skills and confidence are currently being planned.

## IMPLEMENTATION OF DATA SHARING POLICY

To increase the rigor and reproducibility of research, enable data reuse, and promote open science, *JMLA* instituted a data sharing policy in 2019 that requires authors of original investigations, case reports, and special papers to place their underlying data in a repository and include a Data Availability Statement in their manuscript [3, 4]. This step forward served to make *JMLA* the first journal in librarianship to adopt a firm data sharing policy. As expected, some authors have needed reminders or extra guidance toward complying with this policy, but, overall, we have been pleased with how quickly *JMLA* authors have incorporated data sharing into their scholarly publishing work flow.

## ALIGNMENT WITH MEDICAL LIBRARY ASSOCIATION DOMAIN HUBS

The recent transformation of MLA communities defined seven domain hubs organized by areas of professional practice—clinical support, education, global health & health equity, information management, information services, innovation & research practice, and professionalism & leadership—to enable MLA caucuses to collaborate on activities and programs of interest. As part of this transformation, seven members of the *JMLA* editorial team volunteered to serve as liaisons to each MLA domain hub. In addition, other members of the editorial team are currently conducting an analysis of the degree to which *JMLA* articles published over the last ten years align with the MLA domain hubs. The results of this analysis will be described in a future editorial and used to inform future calls for submissions or other *JMLA* initiatives.

## SOCIAL MEDIA PRESENCE

Aided by 3 successive social media editors, *JMLA*'s social media presence has grown substantially over the last 4 years: from 104 to 618 Twitter (@JrnlMedLibAssn) followers and from 567 to 1,220 Facebook (@MLAjournal) followers. We continue to contemplate strategies for utilizing social media to enrich *JMLA*'s reader and author experiences, such as hosting Twitter chats or online journal clubs centered on recently published *JMLA* articles.

## JOURNAL IMPACT FACTOR

**Figure 3:**
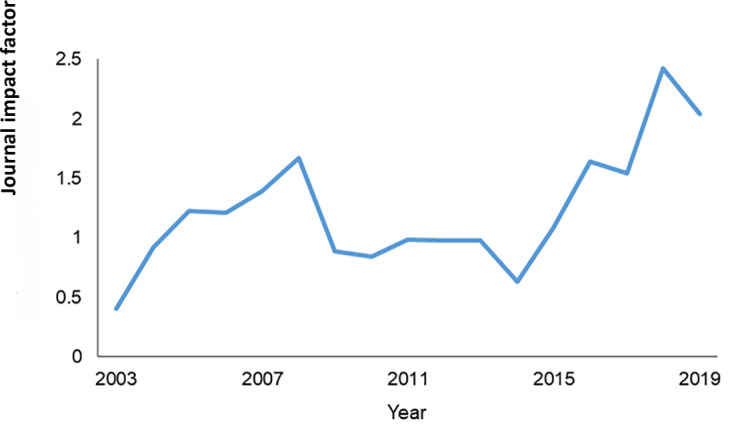
The *Journal of the Medical Library Association*'s (*JMLA*'s) impact factor (Clarivate Analytics) across years

Historically, *JMLA*'s impact factor (Clarivate Analytics) has tended to hover around 1. In recent years, however, our impact factor has crept upward, reaching all-time highs of 2.420 and 2.042 in 2018 and 2019, respectively (Figure 3). At the risk of succumbing to “impact factor mania” [5], we take pride in this indicator, because it suggests that the work we publish is exerting more influence on scholarly discourse and may be having a correspondingly larger impact on the practice of health sciences librarianship.

## LOOKING FORWARD

We are happy with the progress that *JMLA* has made over the past four years and are excited about the future. In the coming year, we plan to create and share new guidelines and resources for peer reviewers, release a call for submissions on topics pertaining to underrepresented MLA domain hubs, implement the CRediT taxonomy for authors to specify contributors' roles in articles, and establish approaches to increasing the diversity of our editorial team and peer reviewers.

In light of national conversations around Black Lives Matter and health equity in response to systemic racism, police violence, and health disparities in the COVID-19 pandemic, we believe it is important to affirm *JMLA*'s commitment to promoting equity in health sciences librarianship and information science. We want to publish articles recognizing and addressing social injustices; attempts to achieve diversity, inclusion, and equity among our workforce and user populations; and other critical perspectives on health sciences librarianship. Acknowledging our positionality as white women who are serving as *JMLA* editors, we believe it is crucial to use our privileged positions to solicit and publish submissions on these topics, particularly those authored by our Black, Indigenous, and People of Color (BIPOC) colleagues. This is an ongoing call for submissions rather than an intention to compile a special issue because we believe that these topics should be steadily woven throughout our thoughts, conversations, research, writing, and practice. We strive to ensure that *JMLA* continues to reflect the cutting edge of theory and the realities of practice while advancing health sciences librarianship in new directions and look forward to bringing you a valuable resource for many years to come.
